# Elevated m 6 A RNA Modifications Associate with Immune Dysregulation and Cancer in People with HIV-1

**DOI:** 10.21203/rs.3.rs-8070380/v1

**Published:** 2026-01-13

**Authors:** Tarun Mishra, Shraddha Tripathi, Jack T. Stapleton, Li Wu

**Affiliations:** University of Iowa; University of Iowa; University of Iowa; University of Iowa

**Keywords:** HIV-1 infection, cancer, people living with HIV-1 (PLWH), N6-methyladenosine (m6A), peripheral blood mononuclear cells, type-I interferon (IFN-I), IFN-responsive genes, antiretroviral therapy

## Abstract

Background: N 6-methyladenosine (m 6 A) modifications of human immunodeficiency virus type 1 (HIV-1) and cellular RNA contribute to viral immune evasion and regulation of host and viral gene expression. We reported elevated RNA m 6 A levels in peripheral blood mononuclear cells (PBMCs) from HIV-1 viremic individuals compared to those on antiretroviral therapy (ART). RNA m 6 A dysregulation has been implicated in many types of cancer. However, the role of m 6 A modifications in HIV-1-associated cancers remains to be investigated. In this study, we aim to address this important question using clinical samples. Methods: We quantified RNA m 6 A levels in PBMCs from 43 de-identified people living with HIV-1 (PLWH), comparing those with cancer (n=15) to those without cancer (n=28). We used enzyme-linked immunosorbent assay (ELISA) to measure RNA m 6 A levels in PBMCs. Using an array of reverse transcription quantitative polymerase chain reaction (RT-qPCR), we performed quantitative transcriptomic analysis of 84 IFN-I-responsive genes in PBMCs. Furthermore, we performed linear regression analyses of cellular RNA m 6 A levels with HIV-1 RNA copies and CD4 + T cell counts in peripheral blood. Results: We found that m 6 A levels of PBMCs were 2.8-fold higher in the cancer group and correlated with expression of m 6 A regulatory genes. Higher m 6 A levels were also associated with increased HIV-1 RNA copies and reduced CD4 + T cell counts. HIV-1 viral load in the cancer group was higher than the non-cancer group. Transcriptomic analysis of 84 IFN-I-responsive genes revealed upregulation of many pro-inflammatory and interferon-stimulated genes in PLWH with cancer. Conclusions: Our findings suggest that HIV-1 infection and cancer microenvironment-mediated m 6 A reprogramming may contribute to chronic immune activation and malignancy in PLWH. Our results also highlight a post-transcriptional mechanism linking HIV-1 persistence to cancer risk.

## Background

The eukaryotic RNAs have more than 170 types of modifications, among which *N*^6^-methyladenosine (m^6^A) is the most abundant internal modification in mRNA and various non-coding RNAs [1, 2]. m^6^A modification influence RNA metabolism by regulating splicing, nuclear export, translation efficiency and transcript stability [2, 3]. The dynamic m^6^A modifications are installed by writer enzymes [methyltransferase like 3/14 (METTL3/14) and Wilms tumor 1-associated protein (WTAP)], removed by erasers [AlkB homolog 5 (ALKBH5) and fat mass and obesity-associated protein (FTO)], and interpreted by reader proteins [YTH domain-containing family 1–3 (YTHDF1–3), collectively called as m^6^A regulatory machinery [4]. The dysregulation of RNA m^6^A modification and its regulatory machinery, including writers, erasers and readers are frequently observed in pathogenesis of various diseases including viral infections and cancers [5–7]. m^6^A modifications have been found on both HIV-1and host transcripts, depicting an intricate role for epitranscriptomic regulation in viral replication, immune evasion, and latency reactivation [8–12]. HIV-1 not only exploits the host m^6^A methylation machinery to enhance its gene expression and replication, but also modulates the expression of m^6^A regulators, leading to broader effects on host immune pathways and gene expression [7].

Our previous study showed that combined antiretroviral therapy (ART) significantly suppresses RNA m^6^A levels in peripheral blood mononuclear cells (PBMCs) from people living with HIV-1 (PLWH) [13]. Despite the success of ART, PLWH exhibit a significantly increased risk of developing certain cancers, including Kaposi’s sarcoma, non-Hodgkin lymphoma, and cervical cancers [14, 15]. This increased cancer risk is partially attributed to chronic immune activation, persistent inflammation, co-infections with oncogenic viruses, and impaired immune surveillance [16]. However, the contribution of RNA modifications, especially m^6^A methylation to oncogenesis in PLWH remains underexplored.

Previous studies have shown the oncogenic roles of several m^6^A regulatory proteins, such as METTL3, METTL14, FTO and YTHDF2 proteins, in promoting proliferation, metastasis and resistance to apoptosis across various cancer types [5, 17–19]. Given the dual role of m^6^A in modulating antiviral immunity and cancer-associated gene expression, it is plausible that aberrant m^6^A signaling may serve as a mechanistic bridge between chronic HIV-1 infection and cancer development. Notably, m^6^A also regulates the expression and function of interferon-stimulated genes (ISGs), which are central to antiviral defense but can paradoxically promote tumorigenesis under chronic activation [20]. This intersection raises the possibility that chronic HIV-1-associated immune activation, combined with epitranscriptomic dysregulation, may influence cancer susceptibility through sustained interferon (IFN) signaling and ISG modulation.

In this study, we investigated the m^6^A RNA methylations in PBMCs from 43 de-identified aging PLWH with or without cancer at average age of 56 and 60, respectively. We found significantly elevated RNA m^6^A levels in PLWH with cancer, which were accompanied by upregulated expression of mRNA encoding the m^6^A writer complex and reader YTHDF1–3 proteins. Furthermore, we observed that the cellular RNA m^6^A levels correlated with higher HIV-1 RNA copy numbers and lower CD4^+^ T cell counts. Importantly, we demonstrated that cancer development in PLWH was associated with aberrant expression of IFN-I-responsive genes, suggesting a link between m^6^A-modulated immune responses and oncogenesis. These findings provide new insights into how m^6^A RNA modifications may contribute to the dysregulation of innate immunity and the development of HIV-1-associated cancers.

## Methods

### Study participants and de-identified PBMCs samples

**Figure 1:**
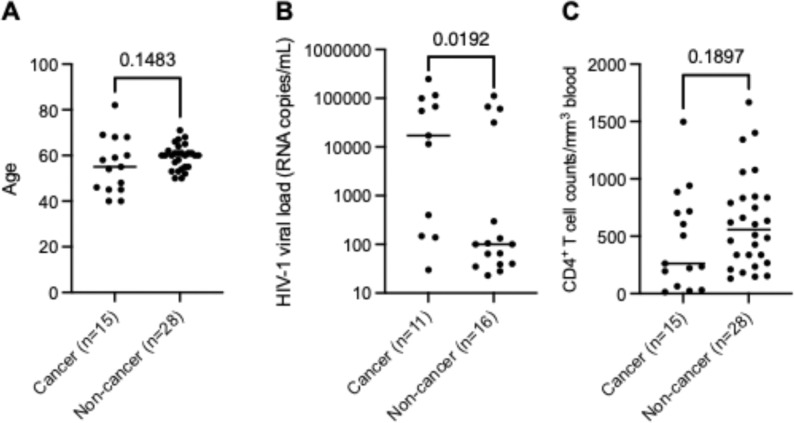
Statistical analyses of three measurable parameters between PLWH with or without cancer. **(A)** age, **(B)** HIV-1 viral load (RNA copies/mL) in non-suppressed PLWH, and **(C)** CD4^+^ T cell counts/mm^3^ blood. **(A-C)** Mann-Whitney U test was used for the statistical analyses. *p* values are labeled on the figures.

PBMCs were obtained from a total of 43 PLWH with cancer (n = 15) or without cancer (n = 28) and de-identified sample details were described in Tables 1 and 2, respectively. Thirteen of the de-identified samples (n = 13) were provided by the AIDS and Cancer Specimen Resource funded by the National Cancer Institute, NIH. Individuals (n = 30) attending the University of Iowa Virology Clinic were invited to participate in this study and, following written informed consent, provided blood samples. The informed consent to participate was obtained from all of the participants in the study. PMBCs were collected from PLWH with a documented history of cancer, or from those without cancer. Blood samples were obtained for the preparation of PBMCs as described [21]. Medical records of those with HIV-1 were reviewed. CD4^+^ T cells count was either obtained at the same visit, or the most recent (within 2 years) in those with over 2 years viral load suppression, and HIV-1 viral load were documented. Plasma HIV-1 viral load was conducted using the COBAS^®^ AmpliPrep/COBAS^®^ TaqMan HIV-1 test (Roche). PBMCs were preserved in liquid nitrogen until use. Statistical analyses of the age, HIV-1 viral load, and CD4^+^ T cells counts between the cancer and non-cancer groups are shown in Fig. 1.

### Cellular RNA isolation and RT-qPCR

Total RNA isolation was performed using TRIzolT^M^ reagent (ThermoFisher Scientific, 15596018) following manufacturers guidelines. The isolated RNA was quantified using a NanoDrop OneC spectrophotometer (ThermoFisher Scientific). DNase-treated RNA (500 ng) served as a template for cDNA synthesis, using the iScript cDNA Synthesis Kit following the manufacturer’s protocol (Bio-Rad, 1708890). qRT-PCR was performed with the iTaqSYBR Green PCR Kit (#1725124, Biorad) in the BioRad CFX96 Real-Time PCR system to evaluate the relative mRNA expression of the m^6^A writer complex genes (*METTL3*, *METTL14*, *WTAP*, *RBM15*, and *VIRMA*), erasers (*ALKBH5* and *FTO*), and readers (*YTHDF1–3*) in PBMCs obtained from PLWH with cancer and without cancer. All reactions were performed in triplicate and normalized with *GAPDH* as a housekeeping gene. The relative gene expression of each sample was calculated using the 2^−ΔΔ*C*t^ formula. The primers for qRT-PCR were described in our previous study [13].

### RNA mA quantification by ELISA

RNA m^6^A ELISA was performed according to the published protocol [12, 13]. Briefly, 200 ng of total RNA per sample was quantified in triplicate. The primary m^6^A antibody solution (ABclonal, A19841) was prepared by diluting 1:10,000 in PBST (PBS with 0.1% Tween 20), and the secondary anti-rabbit IgG solution (Promega, W4011) was prepared by diluting 1:5000 in PBST. Each well was sequentially incubated with 100 μL of primary antibody solution, followed by 100 μL of secondary antibody solution. The signal was developed using 100 μL of 3,3′,5,5′-tetramethylbenzidine substrate (ThermoFisher Scientific, 34021) and quenched with 100 μL of stop solution (2% H_2_SO_4_). Absorbance was measured at 450 nm, and m^6^A levels were quantified relative to a standard curve generated using defined concentrations of m^6^A-modified RNA (EpiGentek, P-9005–96-PC).

## Results

### Elevated RNA m^6^A levels in PBMCs from de-identified PLWH with cancer

To examine the potential effects of HIV-1 infection and cancer status on cellular RNA m^6^A levels *ex vivo*, we obtained PBMCs from 43 de-identified PLWH, including those with various types of cancer (n = 15) and without cancer (n = 28). The informed consent to participate was obtained from all of the participants in the study. The age, gender, HIV-1 plasma viral load, and CD4^+^ T cell counts, cancer type, treatment stage and type, ART regimen, drugs of abuse, use of marijuana of de-identified PLWH with cancer are summarized in Table 1. The age, gender, HIV-1 plasma viral load, CD4^+^ T cell counts, ART regimen, drug of abuse, and use of marijuana of de-identified PLWH without cancer are summarized in Table 2. Only de-identified information is provided in Tables 1–2. Statistical analyses indicated that HIV-1 viral load was higher in participants in the cancer group compared to those in the non-cancer groups, while the age and CD4^+^ T cell counts did not show statistical differences between the two groups (Fig. 1).

**Figure 2:**
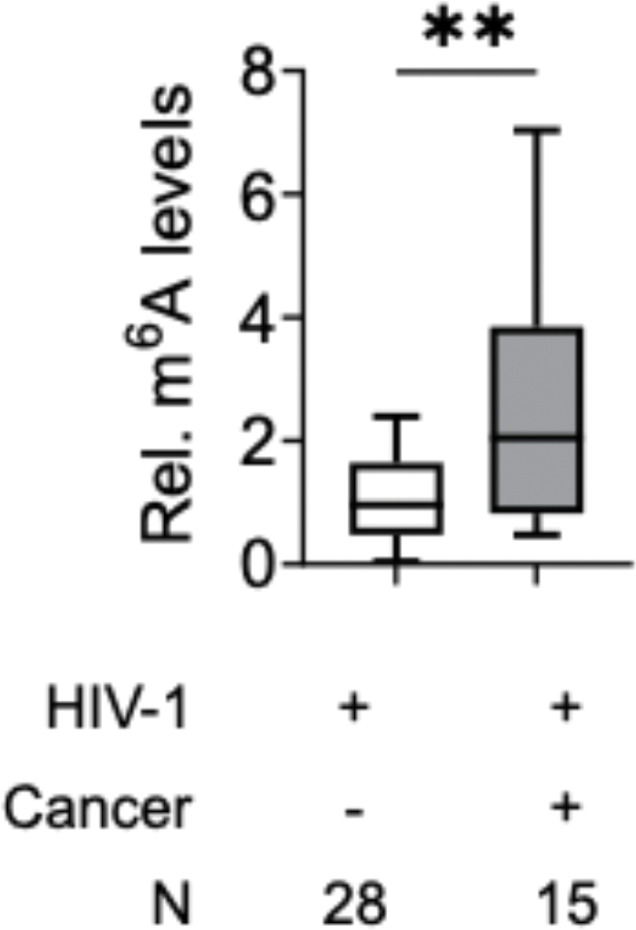
Detection of RNA m^6^A levels in PBMCs from PLWH with or without cancer. Total cellular RNA isolated from PBMCs of 43 PLWH with (n = 15) or without cancer (n = 28) were subjected to m^6^A ELISA (200 ng of RNA/sample). The box plot represents the mean of absolute m^6^A amounts in cancer (+) and cancer (−) PLWH. Non-parametric unpaired Mann Whitney U-test was performed to estimate levels of significance (** *p* ≤ 0.01).

Relative m^6^A levels in RNA from PBMCs of PLWH were quantified using m^6^A ELISA [13]. PLWH with cancer exhibited approximately 2.8-fold higher m^6^A levels than those without cancer (p ≤ 0.01) (Fig. 2). The increased m^6^A levels are also correlated with higher viral load in the cancer group compared to non-cancer group (Fig. 1). This observation extends our previous findings that viremic individuals show elevated m^6^A levels compared to PLWH on ART [13]. These findings suggest a potential link between HIV-1 infection and elevated m^6^A levels, which may contribute to dysregulation of host gene expression and possibly increase the risk of cancer development in PLWH.

### Correlation of cellular RNA mA levels with HIV-1 viral load and CD4 T cell counts

**Figure 3:**
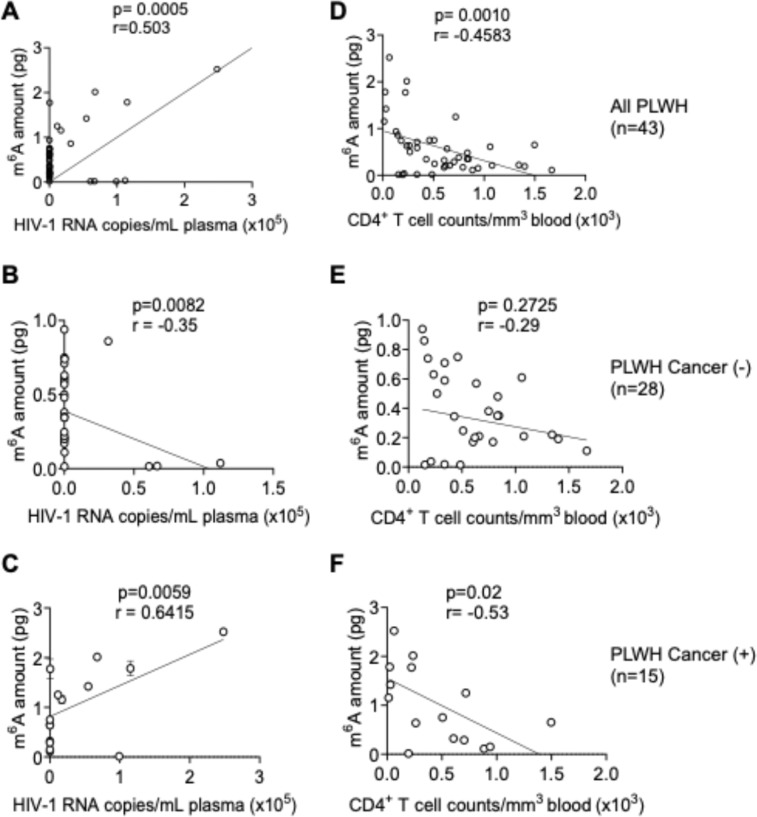
Correlations of m^6^A RNA levels from PBMCs of PLWH with HIV-1 RNA copy numbers and CD4^+^ T cell counts. Simple linear regression analyses of cellular RNA m^6^A amounts and plasma HIV-1 RNA copies/mL (A, B and C) or blood CD4^+^ T cell counts (D, E and F). RNA samples were from PBMCs of **(A, D)** all PLWH (n = 43), **(B, E)** PLWH without cancer (n = 28), and **(C, F)** PLWH with cancer (n =15). Spearman correlation test was used to assess the relationship between the measured parameters. The correlation coefficient and *p* values for each comparison are labeled over each graph**.**

To determine whether RNA m^6^A levels are associated with HIV-1 replication, we analyzed the correlation between m^6^A levels in PBMCs and plasma HIV-1 RNA copies of PLWH. In the combined dataset (n = 43), cellular m^6^A RNA levels showed a significant positive correlation with HIV-1 RNA levels (p = 0.0005; Fig. 3A). When analyzed separately, a significant negative correlation was observed between m^6^A levels and HIV-1 RNA copies in PLWH without cancer (n = 28, p = 0.0082; Fig. 3B), whereas a strong positive correlation was found in PLWH with cancer (n = 15, p = 0.0059; Fig. 3C). These data demonstrate that elevated HIV-1 viral load and cancer tumor microenvironment is associated with increased RNA m^6^A levels in PBMCs, suggesting that HIV-1 viral load and tumor microenvironment in PLWH with cancer enhance cellular RNA m^6^A modifications.

We also assessed the association between cellular m^6^A RNA levels and CD4^+^ T cell counts of PLWH. In the overall cohort, a significant negative correlation was observed (n = 43, p = 0.001; Fig. 3D). This trend remained significant among PLWH with cancer (n = 15, p = 0.02; Fig. 3F), but not in PLWH without cancer (n = 28, p = 0.2725; Fig. 3F). The increased m^6^A levels and the association with CD4^+^ T cell decline in PLWH with cancer may also contribute to heightened cancer risk in PLWH [22, 23].

### Expression of m^6^A regulators genes in PBMCs from PLWH with or without cancer

**Figure 4:**
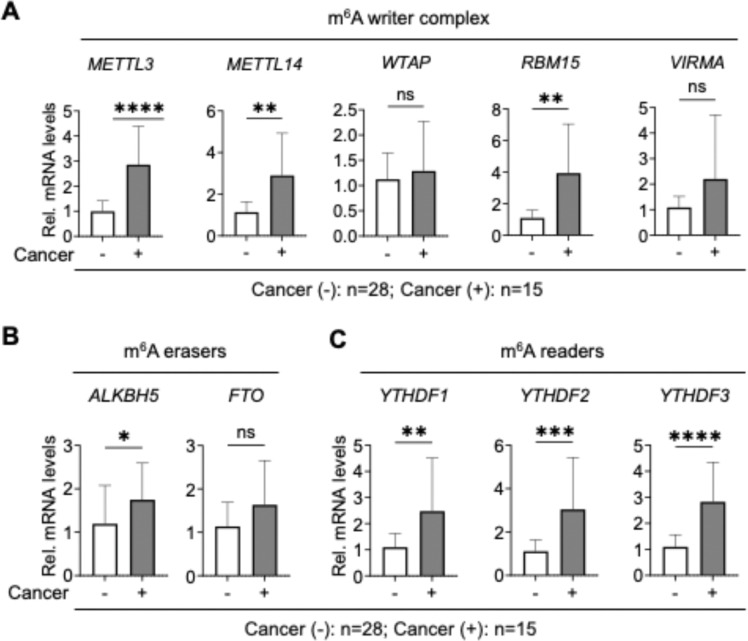
Relative mRNA expression levels of m^6^A regulators in PBMCs from PLWH. RT-qPCR was performed using RNA of PBMCs from the PLWH with cancer (n = 15) or without cancer (n = 28). Relative expression levels were determined by normalizing to *GAPDH* and setting the average expression level in cancer-negative PLWH to 1. Relative mRNA expression levels of **(A)** m^6^A writer complex genes, **(B)** m^6^A eraser genes, and **(C)** m^6^A reader genes. Non-parametric unpaired Mann Whitney U-test was used to estimate the levels of significance (*p < 0.05, **p ≤ 0.01, ***p ≤ 0.001, ****p ≤0.0001, ns, not significant).

To determine whether elevated m^6^A RNA levels in PLWH with cancer are driven by altered expression of m^6^A regulatory genes, we measured the mRNA levels of major genes encoding m^6^A writers, erasers, and readers (Fig. 4). We observed a significant increase in the expression of the core methyltransferase components METTL3, METTL14, as well as the accessory proteins RBM15 and VIRMA, in PBMCs from PLWH with cancer compared to those without cancer (Fig. 4A). In contrast, WTAP, an essential scaffolding subunit of the methyltransferase complex, showed no change in expression, suggesting that its regulatory function may be modulated post-transcriptionally or through other mechanisms [24]. In the m^6^A erasers, we detected a modest increase in *ALKBH5* mRNA expression, while *FTO* mRNA expression remained unchanged according to statistical analysis (Fig. 4B). The mRNA levels of the m^6^A reader proteins YTHDF1–3 were significantly elevated in PLWH with cancer compared to those without cancer (Fig. 4C). These findings suggest that the increased m^6^A RNA methylation levels may be driven by transcriptional upregulation of m^6^A regulatory genes. However, it is important to note that mRNA expression levels do not always correspond to protein abundance or enzymatic activity, and further studies are needed to assess protein-level changes and post-translational regulation.

### Dysregulated expression of IFN-I-responsive genes in PLWH with cancer

**Figure 5:**
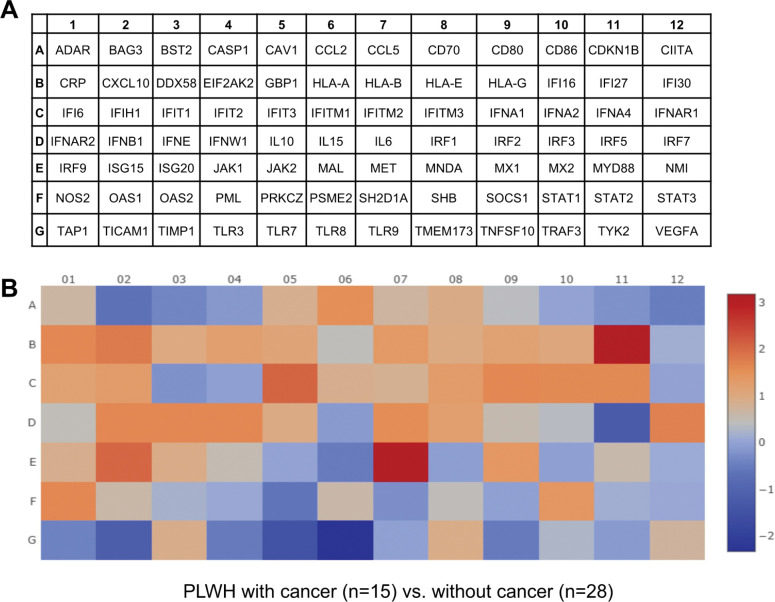
Cancer differentially regulates mRNA expression of IFN-I-responsive genes in PLWH. Relative gene expression was measured in PBMCs from PLWH with cancer (n = 15) or without cancer (n = 28). **(A)** Plate map depicting gene symbols for 84 IFN-I-responsive genes. **(B)** Heat maps show the average differential gene expression in PBMCs of cancer-positive compared with cancer-negative PLWH. The red and blue colors represent upregulated and downregulated genes, respectively. The scales of fold changes are shown next to the legends. Data were analyzed utilizing the web-based RT^2^ profiler PCR array data analysis program (https://geneglobe.qiagen.com/us/analyze).

Given the functional significance of m^6^A modification and importance of IFN-I-associated genes in regulating both HIV-1 infection and cancer [7, 11, 25], we explored the expression of IFN-I-responsive genes in PLWH with cancer compared to individuals without cancer. Using an RT-qPCR array we analyzed the mRNA levels of 84 IFN-I-responsive genes, including interferons, signaling molecules, receptors, ISGs, and IFN-resistance markers. The assay allowed us to estimate the RNA levels of 84 IFN-I associated genes in one plate with respect to five housekeeping genes (Fig. 5A and 5B). Heat maps were generated to depict the average change in gene expression in PLWH with cancer (n = 15) or without cancer (n = 28). Overall, many IFN-I-responsive genes expression levels were upregulated in caner-positive with respect to cancer-negative PLWH (Fig. 5B).

### Significantly regulated IFN-I-responsive gene expression in PLWH with cancer

**Figure 6:**
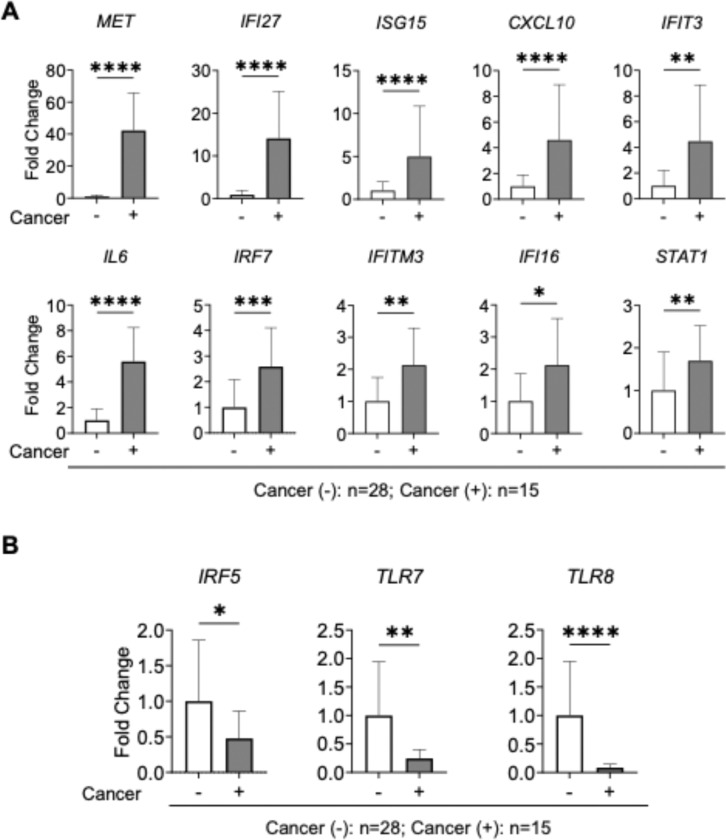
Significantly regulated IFN-I-responsive genes in PBMCs from cancer-positive (+) compared with cancer-negative (−) PLWH. Relative gene expression levels were calculated from the RT^2^ profiler PCR array as shown in Figure 5A. **(A)** Relative mRNA levels of top ten genes (based on fold change) significantly upregulated in PBMCs from cancer (+) relative to cancer (−) PLWH. **(B)** Three genes significantly downregulated in PBMCs from cancer (+) relative to cancer (−) PLWH. **(A** and **B)** Non-parametric unpaired Mann Whitney U-test was used to estimate levels of significance (*p < 0.05, **p ≤ 0.01, ***p ≤ 0.001, and ****p ≤ 0.0001).

Using the IFN-I-responsive gene expression array, we identified a panel of genes significantly upregulated in PLWH with cancer (Fig. 6A). A comparative analysis of the top ten upregulated genes (based on fold change) between cancer-positive and cancer-negative PLWH revealed a distinct transcriptional signature (Fig. 6A). Among these, MET proto-oncogene, receptor tyrosine kinase (*MET*) and IFN-alpha-inducible protein 27 (*IFI27*) mRNA levels were the most significantly elevated in cancer-positive PLWH, exhibiting ~ 32-fold (p = 0.0001) and ~ 14-fold (p = 0.0001) increases, respectively. This observation is consistent with our prior findings that HIV-1 infection induces *MET* and *IFI27* expression in viremic PLWH compared to HIV-1-suppressed individuals on ART [13]. Although the role of IFI27 in HIV-1 infection and progression have been reported [13], its function in context of oncogenesis in PLWH remains to be explored.

MET is involved in cell proliferation, survival, and metastasis [26], and its overexpression has been implicated in various cancers [27], suggesting that its upregulation in the HIV-1 and cancer context may contribute to tumorigenesis. IFI27, a well-known ISG, has been implicated in both viral infection responses [28] and cancer progression through its regulation of apoptosis and immune evasion [29, 30]. Several other IFN-I-responsive genes were also significantly upregulated in cancer-positive PLWH, including *ISG15*, C-X-C motif chemokine ligand 10 (*CXCL10*), interferon-induced protein with tetratricopeptide repeats 3 (*IFIT3*), interleukin 6 (*IL6*), interferon regulatory factor 7 (*IRF7*), interferon-induced transmembrane protein 3 (*IFITM3*), interferon-gamma inducible protein 16 (*IFI16*), and signal transducer and activator of transcription 1 (*STAT1*).

Furthermore, three innate immunity regulatory genes were significantly downregulated (p < 0.05) in cancer-positive PLWH compared to cancer-negative PLWH, including genes encoding interferon regulatory factor 5 (*IRF5*), Toll-like receptor 7 (*TLR7*), and *TLR8* (Fig. 6B). These genes are known to contribute to chronic immune activation, inflammation, and tumor-promoting immune landscapes [31]. Together, these data suggest a dynamic reprogramming of IFN-I signaling in PLWH with cancer, potentially driven by chronic infection and altered m^6^A-mediated gene regulation.

## Discussion

The epitranscriptomic m^6^A RNA modification is the most prevalent internal modification on eukaryotic mRNA and is dynamically regulated by a set of proteins known as m^6^A regulators controlling the fate of RNA. We found that RNA m^6^A levels of PBMCs from PLWH with cancer were higher compared to PBMCs from PLWH without cancer. This suggest that cancers further enhance m^6^A RNA modification in PLWH. These observations align with previous reports [32] that enhanced m^6^A modification levels can serve as a biomarker for both HIV-1 infection and cancer progression.

To explore the connection between m^6^A RNA modification, HIV-1 replication, and cancer status, we performed correlation analysis. We observed a positive correlation between m^6^A levels and HIV-1 RNA copies when data from all PLWH were analyzed collectively (Fig. 3A), supporting previous findings that HIV-1 infection and treatment with recombinant HIV-1 gp120 enhances cellular RNA m^6^A levels [33]. Interestingly, when stratified by cancer status, a positive correlation between m^6^A and viral load was observed in PLWH with cancer, whereas a negative correlation was found in PLWH without cancer. The negative correlation may be influenced by lower average plasma HIV-1 RNA copies in the non-cancer group (mean = 55,813 copies/mL) compared to cancer group (mean = 17,004 copies/mL). The smaller sample size may skew the analysis outcome. Performing the correlation analysis on a large cohort can further support the findings.

We observe a significant negative correlation in CD4^+^ T cell counts and m^6^A RNA levels in combined dataset (n = 43) and in cancer subgroup, while a non-significant trend was observed in non-cancer subgroup. Elevated m^6^A levels might be associated with reduced CD4^+^ T cell recovery in PLWH, particularly in those with cancer. Indeed, individuals with cancer had markedly lower average CD4^+^ counts (460 ± 414) compared to those without cancer (619 ± 399). These findings emphasize the importance of early HIV-1 diagnosis and regular monitoring of both CD4^+^ T cell counts and m^6^A RNA levels to assess cancer risk in PLWH [16, 22, 34, 35].

One limitation in our study is potential confounding variables in the cohort, such as HIV-1 viral load, CD4^+^ T cell counts, ART adherence, and drug use. Our statistical analyses indicate that the difference in the age and CD4^+^ T cell counts is not significant between the cancer and non-cancer groups, while the viral load is significantly higher in the cancer group compared to the non-cancer group (*p* < 0.05). Our previous study showed significant higher RNA m^6^A levels in PBMCs from HIV-1 viremic individuals compared to those on ART [13]. Thus, the higher viral load in the cancer group might contribute to the increased m^6^A RNA levels compared to the non-cancer group. Notably, in the cancer cohort, 6 out of 15 PLWH had not received ART, while in the non-cancer cohort, only 3 out of 28 PLWH had not undergone ART. Therefore, the higher viral load observed in the cancer group may be partly due to the larger proportion of individuals not receiving ART. However, we believe that our results are not solely explained by HIV-1 infection and ART status but are also influenced by the cancer status of the individuals.

Because we observed elevated levels of m^6^A RNA modification in PLWH with cancer compared to PLWH without cancer (Fig. 2), we hypothesized that this increase might be due to altered expression of m^6^A regulatory machinery, including writers, erasers, and readers. We thus analyzed the expression of key m^6^A regulatory genes. We observed a significant upregulation of METTL3 and METTL14 transcripts, the two catalytic components of the m^6^A methyltransferase complex, in cancer samples. Interestingly, mRNA expression of WTAP, a key regulatory subunit that stabilizes and scaffolds the methyltransferase complex [36], remained unchanged (Fig. 4A). This raises the possibility that post-transcriptional or post-translational modifications (PTMs) may regulate WTAP function in HIV-1-associated cancer, requires further studies. We also observed increased expression of RBM15 that guide m^6^A deposition at specific transcript regions. The upregulation of ALKBH5 mRNA expression was unexpected and suggests a more complex regulatory mechanism that might involve substrate specificity, cellular localization, or PTMs.

Due to the limited number of available cells, we could not assess protein expression levels in this study. However, our previous work indicated no significant differences in protein levels of these regulators between PLWH and healthy controls [33]. Thus, the observed increase in m^6^A levels in cancer samples cannot be solely attributed to RNA or protein abundance. It is possible that m^6^A regulatory enzyme activity is modulated by PTMs which are known to influence both writer and eraser functions [37, 38]. Whether such PTM-based modulation contributes to elevated m^6^A levels in PLWH with cancer remains an open question and merits further investigation.

We also found significant mRNA upregulation of *YTHDF1–3*, which encode major members of the YTHDF family of m^6^A reader proteins, in samples from PLWH and cancer (Fig. 4C). These proteins bind m^6^A-modified transcripts and regulate their translation or decay. Previous studies have linked YTHDF proteins to tumorigenesis in various cancers, including hepatocellular carcinoma, leukemia, and glioblastoma [39]. For instance, YTHDF2 promotes degradation of pro-apoptotic or differentiation-associated mRNAs, while, YTHDF1 enhances the translation of growth-promoting mRNAs and has been shown to correlate with poor prognosis in several malignancies. The elevated expression of YTHDF readers may reflect a cellular adaptation to chronic immune activation, viral persistence, or altered m^6^A dynamics induced by HIV-1 or ART. It remains to be determined whether these changes contribute functionally to tumor development or represent a compensatory mechanism in response to altered m^6^A dynamics.

To further explore how m^6^A dysregulation intersects with immune signaling, we analyzed the expression profiles of 84 IFN-I-responsive genes and immune-related genes associated with HIV-1 pathogenesis and cancer progression. We found that mRNA levels of a subset of IFN-I responsive genes, including *MET, IFI27, ISG15, CXCL10, IFIT3, IL6, IRF7, IFITM3, IFI16*, and *STAT1*, were significantly upregulated in cancer samples from PLWH (Fig. 6A). These genes and their encoded proteins play central roles in regulating antiviral immunity and inflammation. Of note, persistent expression of ISGs in PLWH, even under ART, suggests a state of chronic immune activation [40].

Importantly, many of these genes also have established roles in tumorigenesis. For instance, MET is a proto-oncogene implicated in tumor proliferation and metastasis [41]; IL6 is a pro-inflammatory cytokine that supports tumor growth and immune evasion [42]; and STAT1, though typically antiviral, has context-dependent functions in promoting or suppressing tumorigenesis [43]. Additionally, ISG15 and IFI27 modulate apoptosis and immune responses and are upregulated in various cancers and viral infections [44, 45]. Notably, several of these transcripts such as *STAT1, ISG15*, and *CXCL10* have been reported to be regulated by m^6^A modification [20, 46, 47]. This suggests that the m^6^A RNA modification may further shape the chronic inflammatory state observed in these PLWH. A recent study showed that inhibition of METTL3 stimulates IFN-I response by promoting double stranded RNA formation and promoting anti-tumor immunity [48].

Conversely, we observed significant downregulation of IFN-I regulatory or pattern recognition receptor genes, such as IRF5, TLR7 and TLR8 in PLWH with cancer. These genes are vital for sensing viral RNA and activating IFN-I responses [49, 50]. Their reduced expression may represent a mechanism of immune evasion or exhaustion in the tumor microenvironment, potentially contributing to impaired antiviral surveillance and increased cancer susceptibility in PLWH. Due to limited RNA availability from PBMCs in this study, we were unable to assess m^6^A methylation status of individual IFN-I-responsive transcripts. Collection of additional samples will be necessary to determine m^6^A levels and further elucidate its role in regulating RNA stability and translation in aging PLWH with cancer. Inflammation and immunosuppression induced by chronic virus infection and cancer can lead to a dysfunctional immune state [16, 31]. IFN-I-responsive transcripts might play an important role and exert their opposing effects during cancer progression and HIV-1 infection. This potential dual role needs further investigation in future studies.

We believe that the interplay between HIV-1 infection, cancer, and interferon responses is highly complex and likely influenced by multiple, overlapping factors. It is difficult to conclusively determine whether the elevated IFN-I responses observed in our dataset are driven primarily by the presence of cancer, HIV-1 infection, or their combined effect. Importantly, our prior work has shown that the HIV-1 envelope protein gp120 can upregulate m^6^A RNA modification in host cells independently of active viral replication [33]. Additionally, IFN-I transcripts themselves are subject to m^6^A modification, which targets them for degradation and helps fine-tune immune homeostasis [20]. Furthermore, HIV-1 intron-containing RNAs have been shown to activate innate immune sensors and stimulate IFN-I responses. These studies support the possibility that HIV-1 infection can modulate both m^6^A dynamics and IFN-I responses through multiple mechanisms, independent of or in conjunction with cancer. Further functional studies are required to understand the complex mechanism undying the connection between HIV-1 infection, m^6^A RNA modification, and caner progression.

## Conclusions

Collectively, our findings suggest a complex immune landscape in HIV-1-associated cancers characterized by simultaneous overactivation of ISGs and suppression of innate sensing pathways. The interplay between m^6^A modifications and dysregulated interferon signaling may represent a critical axis in the development of HIV-1-associated cancer in aging populations.

## Supplementary Material

Supplementary Files

This is a list of supplementary files associated with this preprint. Click to download.

• MishraTables12updated111225.docx

## Figures and Tables

**Table 1:** Details of PBMC samples from de-identified PLWH with cancer

Participant	Age	Gender	HIV-1 viral load (RNA copies/mL)	CD4 + T cell count/mm^3^ blood	Cancer type ^a^	Cancer stage	Treatment stage and type	ART regimen	Drugs of abuse	Marijuana use
1 ^b^	40	M ^c^	115,037	24	KS ^d^	High Risk	None	None	Unknown	Unknown
2	45	M	67,633	236	KS	Low Risk	None	None	Unknown	Unknown
3	46	M	99,139	195	KS	Low Risk	None	None	Unknown	Unknown
4	45	M	54,766	31	KS	High Risk	None	None	Unknown	Unknown
5	55	M	17,078	14	KS	High Risk	None	None	Unknown	Unknown
6	40	M	248,068	64	KS	High Risk	None	None	Unknown	Unknown
7	48	M	400	223	KS	Low Risk	ART ~ 2.5 mos prior to sample collection	lopinavir/ritonavir and emtricitabine/tenofovir	Unknown	Unknown
8 ^e^	82	M	148	606	Prostate cancer	Remission	Prostatectomy 2006	Biktarvy	No	No
9	54	F ^f^	S ^g^	1,497	Hodgkin’s disease	Remission	Splenectomy, 1980s	Biktarvy	Yes, many	Yes
10	68	M	S	885	Non-melanocytic skin cancer	Cured	Surgical removal	Biktarvy	No	Yes
11	59	M	30	262	KS	Remission	Diagnosed 2012-responded to ART	Biktarvy	Yes	No
12	69	M	139	506	Large B cell lymphoma	Remission	Treated with RCHOP 2023	Biktarvy	No	Yes
13	60	F	S	941	Vulvar cancer, Anal squamous cell × 3	remission, active	Resected 2022, lymph node resection, XRT, Resected 2024–2025	Juluca	No	No
14	58	M	S	703	Anal dysplasia	Removed	Resected 2016	Biktarvy	No	No
15	68	F	11,500	718	Lung cancer	Resected	Adenocarcinoma, resected and XRT 2022–2023	Biktarvy	Yes, remote	No
**Mean ± SD**	**56 ± 12**		**55,813 ± 72,821**	**460 ± 414**						

Only de-identified information is shown in the table. The informed consent to participate was obtained from all of the participants.

a Cancer present or being treated at time of blood acquisition.

b Samples from participants 1–7 were provided by the AIDS and Cancer Specimen Resource funded by the National Cancer Institute, NIH; Cancer stage is based on ACTG Staging for KS using tumor extent, immune status, and systemic illness factors.

c M: male.

d KS: Kaposi sarcoma.

e Samples from participants 8–15 were provided by the University of Iowa HIV Clinic.

f F: female.

g S: HIV-1 RNA suppressed (viral load ≤ 20 copies/mL).

**Table 2:** Details of PBMC samples from de-identified PLWH without cancer

Participant ID	Age	Gender	HIV-1 viral load (RNA copies/mL)	CD4 + T cell count/mm^3^ blood	ART regimen	Drugs of abuse	Marijuana use
1 ^a^	54	M ^b^	40	511	Lamivudine/Zidovudine/Nevirapine	No	No
2	52	M	60,785	154	None	No	No
3	61	F ^c^	100	428	Lamivudine/Zidovudine/Nevirapine	No	No
4	55	M	100	485	Lamivudine/Zidovudine/Efavirenz	No	No
5	61	F	66,714	337	None	No	No
6	62	M	112,030	211	None	No	No
7 ^d^	60	M	S ^e^	661	Biktarvy	Yes - many	Yes
8	53	M	35	338	Genvoya	No	Yes
9	68	M	S	1,400	Dovato	No	Yes
10	66	M	S	267	Juluca	No	Yes
11	65	M	S	749	Triumeq	No	No
12	61	M	S	790	Descovy + Tivicay	No	No
13	60	M	39	130	Biktarvy	Yes	Yes
14	58	F	S	1,667	Cabenuva	No	No
15	57	M	S	835	Dovato	No	No
16	55	M	133	1,059	Dovato	Yes	Yes
17	71	F	S	1,078	Biktarvy	Yes	Yes
18	53	M	S	338	Genvoya	No	Yes
19	67	M	65	604	Biktarvy	No	No
20	50	M	64	846	Biktarvy	No	No
21	61	M	31,500	147	Biktarvy	Yes	Yes
22	50	M	105	235	Biktarvy	No	No
23	60	F	28	634	Descovy + Tivicay	No	No
24	60	M	S	832	Biktarvy	No	No
25	64	M	S	620	Biktarvy	No	Yes
26	61	M	23	460	Odefsey	No	Yes
27	60	M	S	1,343	Biktarvy	Yes	No
28	60	M	296	182	Biktarvy	No	Yes
**Mean ± SD**	**60 ± 5**		**17,004 ± 32,656**	**619 ± 399**			

Only de-identified information is shown in the table. The informed consent to participate was obtained from all of the participants.

a Samples from participants 1–6 were provided by the AIDS and Cancer Specimen Resource funded by the National Cancer Institute, NIH.

b M: male.

c F: female.

d Samples from participants 7–28 were provided by the University of Iowa HIV Clinic.

e S: HIV-1 RNA suppressed (viral load ≤ 20 copies/mL).

## Data Availability

All data generated or analyzed during this study are included in this manuscript for publication.

## Abbreviations

HIV-1: human immunodeficiency virus type 1
PLWH: people living with HIV-1
m^6^A *N*^6^: methyladenosine
PBMCs: peripheral blood mononuclear cells
ART: antiretroviral therapy
IFN-I: type I interferon
ELISA: enzyme-linked immunosorbent assay
RT-qPCR: reverse transcription quantitative polymerase chain reaction
ISGs: interferon-stimulated genes
METTL3/14: methyltransferase like 3/14
WTAP: Wilms tumor 1-associated protein
ALKBH5: AlkB homolog 5
FTO: fat mass and obesity-associated protein
YTHDF1–3: YTH domain-containing family 1–3
